# Ammonia-induced miRNA expression changes in cultured rat astrocytes

**DOI:** 10.1038/srep18493

**Published:** 2016-01-12

**Authors:** Jessica Oenarto, Ayse Karababa, Mirco Castoldi, Hans J. Bidmon, Boris Görg, Dieter Häussinger

**Affiliations:** 1 Clinic for Gastroenterology, Hepatology and Infectious Diseases, Heinrich Heine University, D 40225 Düsseldorf, Germany; 2 Cecile & Oskar Vogt Institute for Brain Research; Heinrich Heine University, D 40225 Düsseldorf, Germany

## Abstract

Hepatic encephalopathy is a neuropsychiatric syndrome evolving from cerebral osmotic disturbances and oxidative/nitrosative stress. Ammonia, the main toxin of hepatic encephalopathy, triggers astrocyte senescence in an oxidative stress-dependent way. As miRNAs are critically involved in cell cycle regulation and their expression may be regulated by oxidative stress, we analysed, whether astrocyte senescence is a consequence of ammonia-induced miRNA expression changes. Using a combined miRNA and gene microarray approach, 43 miRNA species which were downregulated and 142 genes which were upregulated by NH_4_Cl (5 mmol/l, 48 h) in cultured rat astrocytes were found. Ammonia-induced miRNA and gene expression changes were validated by qPCR and 43 potential miRNA target genes, including HO-1, were identified by matching upregulated mRNA species with predicted targets of miRNA species downregulated by ammonia. Inhibition of HO-1 targeting miRNAs which were downregulated by NH_4_Cl strongly upregulated HO-1 mRNA and protein levels and inhibited astrocyte proliferation in a HO-1-dependent way. Preventing ammonia-induced upregulation of HO-1 by taurine (5 mmol/l) as well as blocking HO-1 activity by tin-protoporphyrine IX fully prevented ammonia-induced proliferation inhibition and senescence. The data suggest that ammonia induces astrocyte senescence through NADPH oxidase-dependent downregulation of HO-1 targeting miRNAs and concomitant upregulation of HO-1 at both mRNA and protein level.

Astrocyte dysfunction due to osmotic and oxidative/nitrosative stress is central to the pathogenesis of hepatic encephalopathy (HE)^1^,^2^. Consequences of an increased formation of reactive nitrogen and oxygen species (RNOS) are posttranslational modifications of proteins^3^,^4^,^5^, RNA oxidation^6^ and an altered zinc-dependent gene transcription in astrocytes^7^,^8^. Such alterations may impair synaptic plasticity and trigger disturbances of oscillatory networks in the brain^9^ thereby producing clinical symptoms of HE^10^. More recently it was found that ammonia, a major toxin in the pathogenesis of HE, promotes astrocyte senescence in a RNOS dependent way^11^, which could explain the recently described persistence of cognitive impairment after resolution of overt HE^12^,^13^. However, molecular mechanisms underlying ammonia-induced astrocyte senescence are as yet incompletely understood^11^.

Heme oxygenase 1 gene transcription is highly sensitive to induction by RNOS and therefore often serves as a biomarker for oxidative/nitrosative stress^14^. Heme oxygenase 1 protein (HO-1) degrades heme into carbon monoxide (CO), ferrous iron (Fe II) and biliverdin, which is further metabolized to bilirubin through biliverdin reductase^15^. As CO and biliverdin have strong anti-inflammatory and anti-oxidative properties^16^,^17^, upregulation of HO-1 in neurological diseases was considered as a protective stress response^18^. On the other hand, free intracellular iron may be taken up by mitochondria where it acts as a pro-oxidant^15^. Enhanced RNOS formation due to iron overload in mitochondria may then impair mitochondrial functions and induce cellular senescence^14^,^15^. In line with this, elevated HO-1 protein levels and deranged iron homeostasis are features of the aging brain^19^ and overexpression of HO-1 in astrocytes induces senescence^15^ and is associated with behavioural abnormalities in mice^20^. Upregulation of HO-1 was also shown in ammonia-treated cultured rat astrocytes^21^, in different animal models for HE^21^,^22^,^23^ and in *post mortem* human brain samples of patients with liver cirrhosis and HE^24^. However, whether upregulation of HO-1 contributes to ammonia-induced astrocyte senescence in HE is currently unknown.

MicroRNAs (miRNAs) are small non-coding RNAs (19–23nts) that inhibit gene expression at the post-transcriptional level through bridging the binding of the RNA interfering silencing complex (RISC) to the target sequence localized within the 3′- untranslated regions (3′UTRs) of the target mRNAs^25^. Functionally, the binding of a particular miRNA to its target sequence can either induce degradation or translational inhibition of the bound mRNA^25^. Importantly, as miRNA binding to their target sequences occurs via partial complementarity, individual target mRNAs may be co-regulated by several miRNAs^26^. By this, miRNA activity regulates numerous biological functions such as differentiation^27^ and apoptosis^28^, while the dysregulation of miRNA expression has been linked to different human diseases including neurological disorders^29^. To date the molecular mechanisms regulating miRNA expression at the transcriptional and post-transcriptional level have been only partially characterized. However, recent evidence suggests that the expression of some miRNA species, which were termed “redoximiRs”^30^, is modulated through oxidative/nitrosative stress.

In the present study, we investigated, whether ammonia affects miRNA expression and whether such modulation would affect the transcriptional output in cultured rat astrocytes. By using a microarray screening approach we identified 43 downregulated miRNA species. Importantly, bioinformatics analysis identified an overlap of 43 genes between the miRNA-predicted targets and the upregulated genes in ammonia-exposed astrocytes. Interestingly, 6 miRNAs were predicted to bind to the 3′UTR of HO-1. Ammonia-induced downregulation of HO-1 mRNA-targeting miRNAs upregulates HO-1 at both mRNA and protein levels, which in turn promotes ammonia-induced growth arrest and senescence in cultured rat astrocytes.

## Results

### Ammonia-induced miRNA expression changes in cultured rat astrocytes and identification of potential target genes

**Figure 1:**
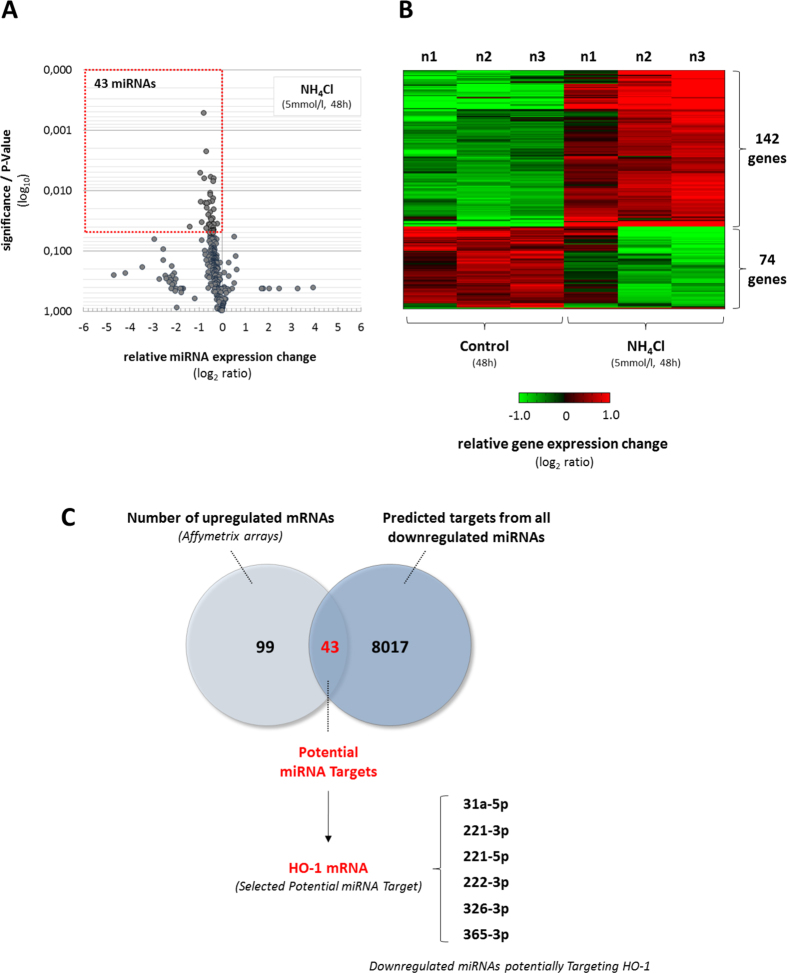
Ammonia-induced miRNA and gene expression changes in cultured rat astrocytes. Cultured rat astrocytes were exposed to NH_4_Cl (5 mmol/l) or were left untreated (control) for 48 h before RNA was isolated and analysed for **(A)** miRNA or **(B)** gene expression changes by Agilent miRNA Array or Affymetrix Rat Gene Array, respectively. **(A)** Volcano plot analysis of miRNA expression changes in NH_4_Cl (5 mmol/l, 48 h)-exposed astrocytes compared to untreated controls. Relative miRNA expression changes with a P-value ≤0.05 were considered statistically significant (see boxed area). **(B)** Illustration of genes with altered expression using a heat map which indicates a stronger or lower expression in red or green, respectively when compared to the median of the expression levels in all samples analysed for a respective gene. Relative gene expression changes of at least two-fold and a P-value ≤0.05 were considered statistically significant. **(C)** Illustration of upregulated mRNA species and miRWALK-predicted mRNA targets from all downregulated miRNAs in NH_4_Cl (5 mmol/l, 48 h)-exposed astrocytes in a Venn diagram depicting 43 mRNAs as potential miRNA targets. Identification of heme oxygenase 1 (HO-1) as a potential target of 6 miRNA species downregulated in NH_4_Cl (5 mmol/l, 48 h)-exposed astrocytes. Data are from 3 independent experiments.

By performing a broad screening approach using microarray technique we analysed, whether ammonia induces gene expression changes in astrocytes in a miRNA-dependent way. NH_4_Cl (5 mmol/l, 48 h) downregulated the expression of 43 out of a total of 336 miRNA species analysed in cultured rat astrocytes (Fig. 1A; Suppl. Fig. 1). Remarkably, no significantly upregulated miRNA species were detected in NH_4_Cl (5 mmol/l, 48 h)-exposed astrocytes as compared to untreated controls (Fig. 1A; Suppl. Fig. 1).

Using Affymetrix rat gene arrays we found that NH_4_Cl (5 mmol/l, 48 h) significantly affected the expression of about 1% of microarray-represented genes when compared to untreated controls (Fig. 1B; Suppl. Fig. 1). With this approach a total of 216 genes, with altered expression level more than 2.0-fold at a statistical significance level of p<0.05, were identified in NH_4_Cl (5 mmol/l, 48 h)-exposed astrocytes. Specifically, the expression of 142 genes and 74 genes was found to be increased and decreased, respectively (Fig. 1B; Suppl. Fig. 1). However, as this study intended to correlate miRNA to mRNA expression we focused solely on the 142 upregulated genes.

Gene expression changes identified by array analysis in NH_4_Cl (5 mmol/l, 48 h)-exposed astrocytes were verified by quantitative polymerase chain reaction. Using qPCR we confirmed upregulation of 22 out of 26 selected genes in NH_4_Cl (5 mmol/l, 48 h)-exposed cultured astrocytes (Suppl. Fig. 2) strengthening the validity and robustness of the array measurement. The data indicate that ammonia induces specific changes in the transcriptome and miRnome in astrocytes and identify a subset of upregulated genes as potential targets of miRNAs downregulated by ammonia.

To identify correlations between the two data sets, miRNA target prediction databases miRWALK and RNA22 were queried to identify all the predicted target genes for downregulated miRNAs in NH_4_Cl (5 mmol/l, 48 h)-exposed astrocytes (Fig. 1A, Suppl. Fig. 1A). Importantly, from 8060 putative targets, 43 were found to overlap with genes upregulated in NH_4_Cl treated astrocytes (Fig. 1C). For further analysis we selected from the 43 potential miRNA target genes heme oxygenase 1 (HO-1), which was predicted by mirWALK analysis as a target for 5 individual miRNA species downregulated by NH_4_Cl (5 mmol/l, 48 h, Suppl. Table 1). By using RNA22 we additionally found 4 predicted binding sites in the HO-1 mRNA sequence for rno-miR-221-5p, which also became donwregulated by NH_4_Cl (5 mmol/l, 48 h) in cultured astrocytes (Fig. 1C, Suppl. Table 2). These correlations suggest that ammonia-induced miRNA-downregulation might play a role in the modulation of HO-1 expression.

### Validation and pharmacological characterisation of ammonia-induced miRNA expression changes in cultured rat astrocytes

**Figure 2:**
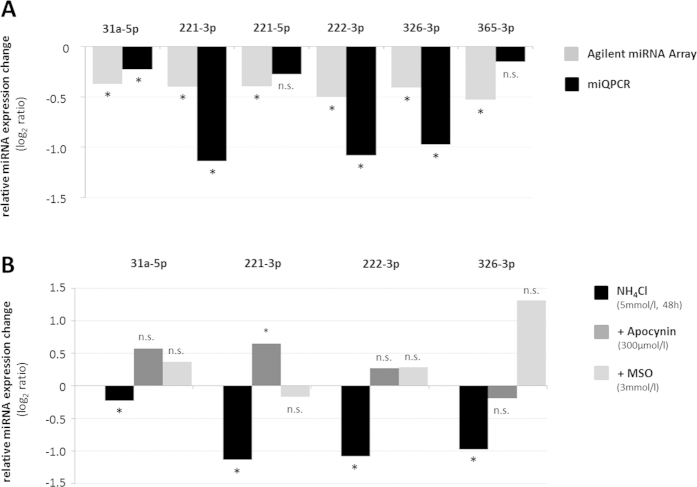
Validation of miRNA expression changes and their dependence on glutamine synthesis and NADPH-oxidase activation in ammonia-exposed astrocytes. **(A/B)** Cultured rat astrocytes were exposed to NH_4_Cl (5 mmol/l) or were left untreated (control) for 48 h before RNA was isolated and analysed for miRNA expression changes by Agilent miRNA Array **(A)** or miQPCR **(A/B)** as described in materials and methods. **(A)** Validation of miRNA expression changes by miQPCR. **(B)** Glutamine synthetase and NADPH-oxidase dependence of miRNA expression changes in NH_4_Cl (5 mmol/l, 48 h)-exposed astrocytes. Where indicated astrocytes were treated with MSO (3 mmol/l) or apocynin (300 μmol/l). *statistically significantly different to the respective control (untreated or inhibitor-treated). n.s.: not significantly different to the respective control (untreated or inhibitor-treated). Data are represented as log_2_ ratios from 3–10 independent experiments.

miRNA expression changes identified by array analysis in NH_4_Cl (5 mmol/l, 48 h)-exposed astrocytes (Fig. 1A) were verified by miRNA-specific quantitative polymerase chain reaction (miQPCR) analysis as described recently^31^. Using miQPCR, we confirmed the significant downregulation of 4 out of 6 miRNAs predicted to target HO-1 mRNAs, while a strong tendency towards downregulation was found for rno-miR-221-5p and -365-3p, however without reaching statistical significance (Fig. 2A). We further confirmed downregulation of 9 additional miRNA species by NH_4_Cl (5 mmol/l, 48 h) in astrocytes which were not predicted to target HO-1 mRNA (Suppl. Fig. 3A).

In order to test for a role of NH_4_Cl (5 mmol/l, 48 h)-induced glutamine formation on miRNA expression changes, astrocytes were treated with the glutamine synthetase inhibitor methionine sulfoximine (MSO, 3 mmol/l). As shown in Fig. 2B, MSO administration fully prevented NH_4_Cl (5 mmol/l, 48 h)-induced downregulation of several miRNAs (Fig. 2B and Suppl. Fig. 3B) including four out of six miRNAs (i.e. rno-miR-31a-5p, -221-3p, -222-3p and -326-3p) predicted to target HO-1 gene.

Ammonia is known to induce the formation of reactive oxygen species (ROS). A role of ROS for NH_4_Cl (5 mmol/l, 48 h)-induced miRNA expression changes was analysed using the NADPH oxidase (NOX)-inhibitor apocynin (300 μmol/l). Apocynin (300 μmol/l) fully prevented NH_4_Cl (5 mmol/l, 48 h)-induced downregulation of 4 miRNA species predicted to target HO-1 (e.g. rno-miR-31a-5p, -221-3p, -222-3p and -326-3p) as well as downregulation of 9 additional miRNA species analysed (Suppl. Fig. 3B).

The data suggest that miRNA expression levels in cultured rat astrocytes are, at least in part, affected by ammonia in a glutamine synthesis- and NADPH oxidase-dependent manner.

### HO-1 mRNA and proteins levels are upregulated in response to the inhibition of miR-31a-5p, -221-3p, -221-5p, -222-3p, -326-3p or -365-3p

**Figure 3:**
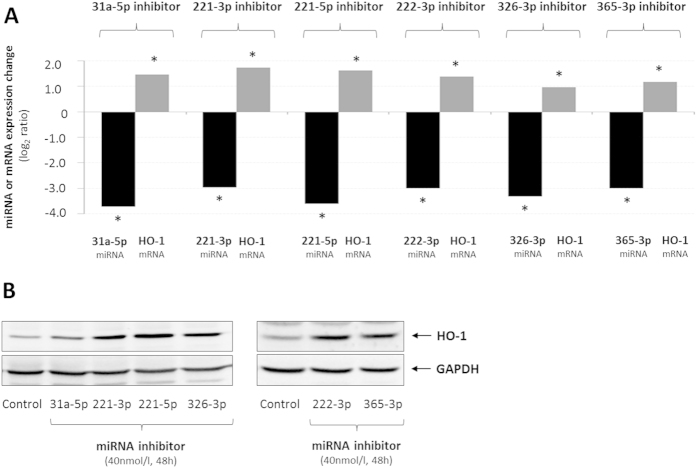
Inhibition of miRNAs predicted to target HO-1 and being downregulated in ammonia-exposed astrocytes elevate HO-1 mRNA and protein levels. Cultured astrocytes were transfected either with inhibitors specifically targeting the indicated miRNA species or without inhibitor (control) before total RNA (**A**) or protein (**B**) was isolated. (**A**) Validation of miRNA inhibition by inhibitors specifically targeting the indicated miRNA species using miQPCR and effect of miRNA inhibition on (**A**) HO-1 mRNA and (**B**) protein levels as analysed by realtime-PCR and Western-blot, respectively. *statistically significantly different compared to control-transfected astrocytes. Data are represented as log_2_ ratios from 5–9 independent experiments.

Bioinformatic prediction suggests that HO-1 is a potential target gene of rno-miR-31a-5p, -221-3p, -221-5p, -222-3p, -326-3p and -365-3p (Suppl. Tables 1, 2). To evaluate the interaction between these miRNAs and HO-1, primary astrocytes were transfected with miRIDIAN® small hairpin miRNA inhibitors and HO-1 expression was measured both at mRNA and protein levels (Fig. 3). As shown in Fig. 3A, the expression of miR-31a-5p, -221-3p, -221-5p, -222-3p, -326-3p or -365-3p is strongly reduced in the astrocytes transfected with the corresponding miRIDIAN® inhibitors (40 nmol/l, each) and concurrently upregulated HO-1 at both mRNA (Fig. 3A) and protein (Fig. 3B) levels. Remarkably, the simultaneous transfection of miRNAs rno-miR-31a-5p, -221-3p, -221-5p, -222-3p, -326-3p and -365-3p increased HO-1 mRNA levels in a synergistic manner as compared to the individual treatment (Suppl. Fig. 4).

To further evaluate the effect of the miRNA inhibition on astrocyte transcriptome, the expression of putative target genes for rno-miR-31a-5p, -221-3p and -326-3p was measured by qPCR. Importantly, these genes, which were upregulated in cultured astrocytes following NH_4_Cl (5 mmol/l, 48 h) administration (Suppl. Fig. 2), were also upregulated in miRIDIAN® transfected astrocytes (Suppl. Fig. 5).

These data indicate that rno-miR-31a-5p, -221-3p, -221-5p, -222-3p, -326-3p and -365-3p are able to modulate HO-1 expression, by either directly targeting HO-1 3′UTR or indirectly by inhibiting the expression of an unknown activator of HO-1 expression or by a combination of both mechanisms.

### Inhibition of miRNAs predicted to target HO-1 impairs astrocyte proliferation

**Figure 4:**
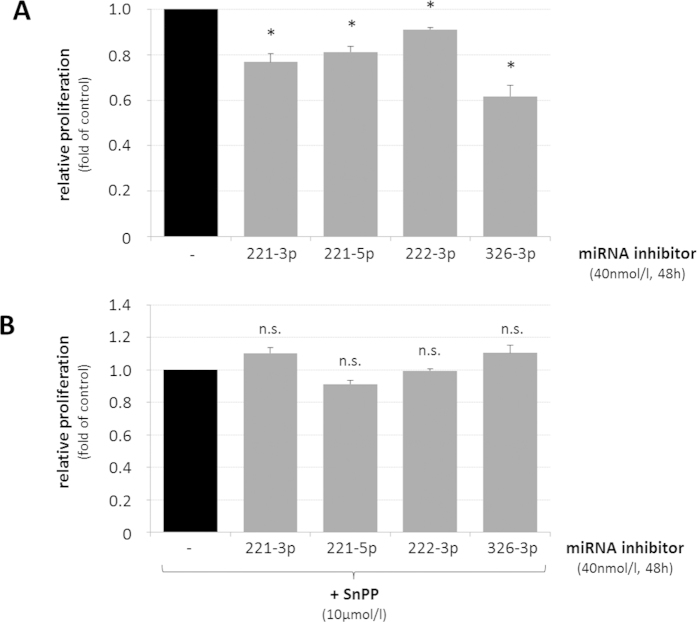
Inhibition of HO-1 mRNA targeting miRNAs impairs astrocyte proliferation in a HO-1-dependent way. Cultured rat astrocytes were transfected with either miRNA inhibitors targeting rno-miR-221-3p, rno-221-5p, 222-3p or -326-3p or without inhibitor (control) and DNA content was quantified 48 h after seeding of the cells by fluorimetric detection of Hoechst34580 fluorescence as described in materials and methods. Fluorescence intensities measured in miRNA inhibitor-treated astrocytes are given relative to transfection control. **(A)** Effect of miRNA inhibition on astrocyte proliferation. **(B)** Effect of HO-1 inhibition by tin protoporphyin IX (SnPP, 10 μmol/l) on proliferation in miRNA inhibitor-treated astrocytes. *statistically significantly different compared to transfection controls. n.s.: not statistically significantly different as compared to SnPP-treated astrocytes. Data are from 3–4 independent experiments.

Upregulation of HO-1 was recently linked to proliferation inhibition and senescence in neurodegenerative diseases^15^. We therefore tested, whether upregulation of HO-1 through inhibition of individual miRNAs predicted to target HO-1 mRNA affected astrocyte proliferation. As shown in Fig. 4A, inhibition of miR-221-3p, -221-5p, -222-3p or -326-3p significantly impaired proliferation of cultured rat astrocytes 48 h after seeding. Conversely, blocking expression of HO-1 by tin-protoporphyrin IX (SnPP, 10 μmol/l), completely prevented growth inhibition as induced by the respective miRNA inhibitors (Fig. 4B).

The presented data show that upregulation of HO-1 expression resulting from the inhibition of individual miRNAs predicted to target HO-1 mRNA (i.e. rno-miR-221-3p, -221-5p, -222-3p or -326-3p), is able to trigger HO-1 dependent growth inhibition in cultured rat astrocytes.

### Role of HO-1 for ammonia-induced astrocyte senescence and growth inhibition

**Figure 5:**
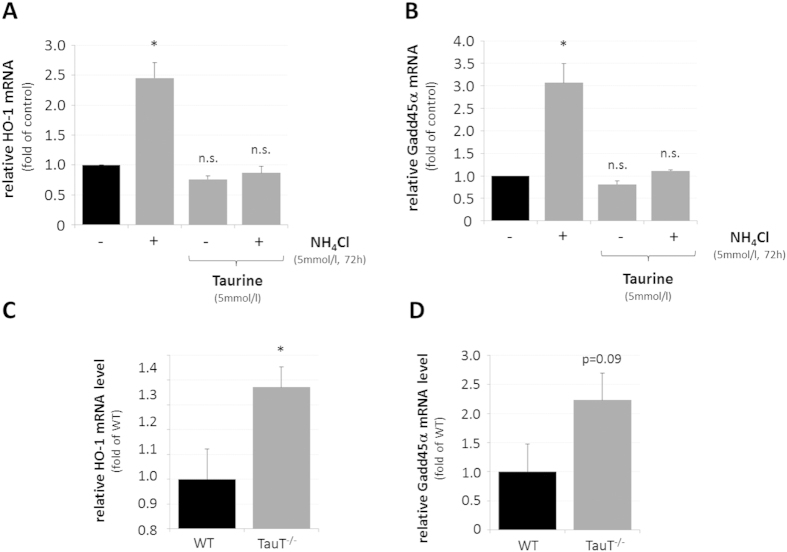
Effect of taurine on HO-1 and Gadd45α mRNA levels in ammonia-exposed cultured astrocytes and in mouse cerebral cortex. Cultured astrocytes were exposed to NH_4_Cl (5 mmol/l) or were left untreated (control) for 72 h before RNA was isolated and analysed for HO-1 **(A)** or Gadd45α **(B)** mRNA levels by realtime-PCR. Where indicated, astrocytes were treated with taurine (5 mmol/l, 16 h pretreatment). Quantification of **(C)** HO-1 or **(D)** Gadd45α mRNA levels by realtime-PCR in wildtype (WT) and taurine transporter knockout (TauT^−/−^) mice. *statistically significantly different compared to the respected control. n.s.: not statistically significantly different. Data are from 3–10 independent experiments.

To evaluate whether the growth inhibition observed in response to miRNA inhibition was effectively associated with HO-1 upregulation, a role of HO-1 for the recently described ammonia-induced astrocyte senescence^11^ was analysed using the osmolyte, antioxidant and chaperone taurine, which was shown to prevent ammonia-induced upregulation of HO-1 in astrocytes^21^. As shown in Fig. 5, taurine (5 mmol/l) prevented the NH_4_Cl (5 mmol/l, 72 h)-induced upregulation of HO-1 mRNA (Fig. 5A) and of growth arrest and DNA-damage-inducible 45a (Gadd45α) mRNA, which is a surrogate marker for senescence (Fig. 5B). In line with this, increased HO-1 (Fig. 5C) and Gadd45α (Fig. 5D) mRNA levels are also observed in cerebral cortex of mice lacking the taurine transporter (TauT) which were reported to have strongly reduced taurine levels in brain^32^.

**Figure 6:**
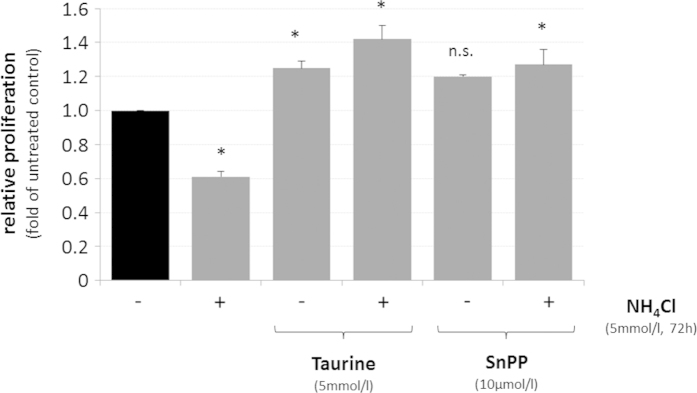
Effect of taurine and HO-1 inhibition on ammonia-induced proliferation inhibition in cultured rat astrocytes. Cultured rat astrocytes were exposed to NH_4_Cl (5 mmol/l) or were left untreated (control) for 72 h in the presence or absence of taurine (5 mmol/l, 16 h pretreatment) or tin protoporphyrin IX (SnPP, 10 μmol/l, 30 min pretreatment). DNA content was quantified by fluorimetric detection of Hoechst34580 fluorescence as described in materials and methods and fluorescence intensities found under the different experimental treatments are given relative to the untreated control. *statistically significantly different compared to untreated controls. n.s.: not statistically significantly different. Data are from 3 independent experiments.

A role of HO-1 for ammonia-induced growth inhibition was analysed using taurine (5 mmol/l) and the HO-1 inhibitor SnPP (10 μmol/l). As shown in Fig. 6, preventing either upregulation of HO-1 by taurine or blocking HO-1 activity by SnPP (10 μmol/l) completely prevented NH_4_Cl (5 mmol/l, 72 h)-induced growth inhibition in cultured rat astrocytes.

The data indicate that HO-1 promotes ammonia-induced senescence and growth inhibition in cultured rat astrocytes, suggesting that the observed ammonia-induced downregulation of miRNAs predicted to target HO-1 could modulate HO-1 expression in astrocytes.

## Discussion

By performing a joint miRNA and gene expression array as well as bioinformatic analysis approach, we identified for the first time ammonia-induced miRNA expression changes and potential miRNA target genes in cultured rat astrocytes (Fig. 1A,B). Ammonia-induced miRNA and gene expression changes identified by microarray analyses were further validated by qPCR reaction (Fig. 2A; Suppl. Figs 2 and 3A). Such a technical approach is required to functionally evaluate consequences of miRNA expression changes in a specific experimental setting, as both, array analysis and bioinformatic miRNA target gene predictions are expected to produce large rates of false positive genes and miRNA targets, respectively^33^.

Here we identified a total of 43 miRNAs downregulated by ammonia in the astrocytes (Fig. 1A). Interestingly, no significantly upregulated miRNA species were detected in ammonia-exposed astrocytes (Fig. 1A; Suppl. Fig. 1). The reason for this is currently unclear. However, selective downregulation of miRNAs in ammonia-treated astrocytes does not reflect ammonia-toxicity or astrocyte death as evidenced by lack of propidium iodide (PI) staining (Suppl. Fig. 6A), absence of terminal deoxynucleotidyl transferase dUTP nick end labeling (TUNEL) immunoreactivity (Suppl. Fig. 6B) and unchanged lactate dehydrogenase (LDH) activity in the supernatant (Suppl. Fig. 6C) in astrocytes treated for up to 72 h with NH_4_Cl (5 mmol/l).

Bioinformatic analysis identified an overlap of 43 genes between the predicted targets of miRNAs downregulated in response to ammonia-administration (e.g. 8060 genes) and the genes which were upregulated by ammonia in cultured astrocytes (i.e. 142 genes; Fig. 1B,C). Interestingly, most of these genes were predicted as putative targets of multiple miRNA species downregulated by ammonia (data not shown), which may indicate that ammonia affects regulatory miRNA networks in the astrocytes.

Ammonia-induced miRNA downregulation was largely prevented by NADPH oxidase inhibition and the glutamine synthetase inhibitor MSO (Fig. 2B; Suppl. Fig. 3B) which both block ammonia-induced oxidative stress^11^,^34^. Therefore, indicating that the miQPCR-validated miRNAs (i.e, 31a-5p, -221-3p, -222-3p and -326-3p) are new members of the redoximiR family^30^ further strengthening the important role of oxidative stress in the pathogenesis of HE^35^.

Interestingly, potential target genes of some ammonia-regulated miRNAs [e.g. Gadd45α, Serpine1, cAMP response element-binding protein 3I1 (Creb3I1) and Myc] were enriched for biological processes implicated in cellular senescence which recently was shown to be induced by oxidative stress in ammonia-exposed cultured astrocytes and suggested to play a major role for the pathogenesis of HE^11^. However, the molecular mechanisms by which ammonia induces senescence in astrocytes are only partially understood^11^.

The present study now suggests, that ammonia inhibits *in vitro* astrocyte proliferation, which represents a surrogate marker for senescence, also through the downregulation of a specific subset of miRNAs (Figs 2A and 4A) that are predicted to target heme oxygenase 1 mRNA (HO-1; Figs 1C and 3A,B; Suppl. Tables 1, 2). In line with previous data^21^ our study confirmed upregulation of HO-1 mRNA (Suppl. Fig. 2) and HO-1 protein (Suppl. Fig. 7) by ammonia and shows that individual inhibition of miRNAs rno-miR-31a-5p, -221-3p, -221-5p, -222-3p, -326-3p or -365-3p upregulates HO-1 at both mRNA and protein levels (Fig. 3A,B). Interestingly, these miRNAs are predicted to bind to different regions of HO-1 3′UTR. In line with this, inhibition of miRNAs rno-miR-31a-5p, -221-3p, -221-5p, -222-3p, -326-3p and -365-3p increased HO-1 mRNA levels in a synergistic manner (Suppl. Fig. 4) further strengthening the view that these miRNAs directly or indirectly modulate HO-1 expression.

In line with an oxidative stress- and redoximiR-dependent upregulation of HO-1, blocking ammonia-induced redoximiR downregulation either by NADPH-oxidase inhibition or by the glutamine synthetase inhibitor MSO (Fig. 2B) significantly inhibited upregulation of HO-1 mRNA by ammonia (Suppl. Fig. 8). This latter finding is at variance with a previous study^21^, probably due to a lack of quantification, a lower detection accuracy provided by the Northern-blot technique as compared to qPCR (this paper) as well as due to differences in the time points analysed (48 h vs 72 h, this paper).

As MSO inhibits ammonia-induced astrocyte swelling^36^ and HO-1 mRNA was also upregulated after hypoosmotic astrocyte swelling^21^, the findings of the present study may suggest that ammonia downregulates redoximiRs at least in part through induction of astrocyte swelling. Indeed, hypoosmotic astrocyte swelling significantly downregulated the expression of two miRNAs predicted to target HO-1 (e.g. rno-miR-221-5p and -326-3p; Suppl. Fig. 9A) and increased HO-1 mRNA levels (Suppl. Fig. 9B).

In line with findings showing that overexpression of HO-1 in cultured astrocytes induces senescence^15^, upregulation of HO-1 either by ammonia (Suppl. Fig. 2), hypoosmolarity (Suppl. Fig. 9B) or by inhibition of rno-miR-221-3p, -221-5p, -222-3p or -326-3p (Fig. 3A,B) impairs astrocyte proliferation (Figs 4 and 6; Suppl. Fig. 9C). Importantly, proliferation inhibition by miRNA-inhibitors rno-miR-221-3p, -221-5p, -222-3p or -326-3p was prevented by SnPP, suggesting a role of HO-1 for miRNA-dependent anti-proliferative effects (Fig. 4B). In line with a role of HO-1 for inhibition of astrocyte proliferation, blocking upregulation of HO-1 mRNA (Suppl. Fig. 8) either by inhibition of NADPH oxidase or by treating the astrocytes with the glutamine synthetase inhibitor MSO fully prevented ammonia-induced astrocyte senescence^11^.

Overexpression of HO-1 was shown to induce astrocyte senescence through RNOS-dependent inhibition of mitochondrial functions^15^. In line with this, there is emerging evidence for mitochondrial dysfunction in hepatic encephalopathy such as induction of the mitochondrial permeability transition^37^, impaired mitochondrial energy metabolism^38^,^39^ and increased mitochondrial ROS formation^11^ which was seen as a consequence of glutamine hydrolysis and subsequent intra-mitochondrial ammonia formation^40^.

Interestingly, mitochondrial dysfunction^41^, accelerated aging and senescence^42^ (this paper, Fig. 5D) as well as increased cerebral HO-1 mRNA expression (Fig. 5C) are features of TauT knockout mice. Moreover, taurine administration prevented upregulation of HO-1 and Gadd45α (Fig. 5A,B) as well as proliferation inhibition (Fig. 6) by ammonia in astrocytes. These findings raise the possibility that HO-1 may contribute to mitochondrial dysfunction in HE. However, further studies are required to clarify the significance of HO-1 for mitochondrial dysfunction and astrocyte senescence in HE.

Interestingly, a very recent study found that overexpression of HO-1 in cultured astrocytes changes miRNA expression levels^43^. Thus, it is reasonable to speculate that HO-1 is not only subject to regulation by miRNAs (this paper) but also triggers miRNA expression changes as part of a complex regulatory network. Interestingly, except for miRNAs rno-miR-187 and -181a, miRNA expression changes induced by overexpression of HO-1^43^ differed from those found in ammonia exposed astrocytes in the present study (data not shown). This may suggest that different kinetics and/or additional factors may contribute to miRNA and gene expression changes in ammonia-exposed astrocytes.

Currently, there is only very little information on changes in the miRnome in the brain in HE. For instance, the only published study to date described miRNA expression changes in the cerebral cortex of mice with azoxymethane (AOM)-induced acute liver failure by using a microarray approach, however, without performing validation of microRNA expression as well as predicted target genes^44^.

Taken together, the present study suggests an important role of ROS-mediated miRNA downregulation and upregulation of HO-1 for ammonia-induced astrocyte senescence (Suppl. Fig. 10).

Further research is required to clarify whether HO-1 expression is directly or indirectly regulated by ammonia-induced changes in miRNA expression and to fully understand the functional significance of changes in the astrocyte miRnome for the pathogenesis of hepatic encephalopathy.

## Methods

### Materials

L-methionin-S-sulfoximine was purchased from Sigma-Aldrich (Deisenhofen, Germany). NH_4_Cl and apocynin were from Merck (Darmstadt, Germany). Cell culture media (DMEM, 1000 mg/l D-glucose, GlutaMAX^TM^) was from Life Technologies (Invitrogen, Karlsruhe, Germany). Fetal calf serum was from Lonza (Cologne, Germany). The polyclonal antibodies against heme oxygenase 1 were from Stressgen® (Victoria, Canada) and the monoclonal antibody raised against glyceraldehyde-3-phosphate dehydrogenase (Gapdh) was from Biodesign International (Cologne, Germany).

### Preparation, cultivation and experimental treatment of rat brain astrocytes

All experimental protocols involving animals were approved by and conducted in accordance with the guidelines of the University of Düsseldorf Institutional Animal Care and Use Committee. Primary rat astrocytes were prepared from the cerebral cortex of newborn Wistar rats (E1–3) as described earlier^3^ and cultured for 4–6 weeks in DMEM (1000 mg/l D-Glucose, GlutaMAX^TM^) containing 10% fetal calf serum (FCS). For experimental treatment, growth medium was replaced by FCS-free DMEM (1000 mg/l D-Glucose, GlutaMAX^TM^).

Astrocytes were treated for 48 or 72 h with 5 mmol/l NH_4_Cl, an ammonia concentration found in brains of animals with experimental hepatic failure^45^ and which is not toxic to cultured astrocytes (Suppl. Fig. 6A–C).

In order to analyse the effects of cell swelling on miRNA and gene expression and proliferation, astrocytes were treated with hypoosmotic (205mosmol/l) cell culture media (DMEM with reduced NaCl concentration, Invitrogen, Karlsbad). As for control, astrocytes were treated with normoosmotic (320mosmol/l) cell culture media (DMEM, 1000 mg/l D-Glucose, GlutaMAX^TM^, Invitrogen, Karlsruhe). Osmolarities of cell culture media were tested using an osmometer (Osmomat030, Gonotec, Berlin, Germany).

### Western-blot analysis

Western-blot analysis was performed as described earlier^11^. At the end of the experimental treatment, protein was purified from cultured astrocytes and protein concentrations of the individual samples were determined by the BioRad protein assay (BioRad, Munich, Germany). Proteins were separated by polyacrylamide gel electrophoresis (12% PAA) and transferred onto nitrocellulose membrane by semi-dry protein transfer. Nitrocellulose membranes were incubated in bovine serum albumin (BSA, 10%) and immunodetection was performed using antibodies against heme oxygenase 1 (HO-1, pAb, 1:1000) or glyceraldehyde-3-phosphate dehydrogenase (mAb, 1:5000), respectively. Primary antibodies were detected using secondary anti-mouse or anti-rabbit antibodies coupled to horseradish peroxidase (1:10000, 2 h, RT), respectively. After blots were washed thrice, peroxidase activity was detected by chemiluminescence (Western-Lightning^TM^, Perkin Elmer, Waltham, USA). Image acquisition was performed using the Kodak Image Station 4000MM and the Kodak Molecular Imaging Software (v.4.0.3).

### RNA isolation and qPCR analysis of mRNA expression

Realtime-PCR was carried out as described earlier^11^. In brief, total RNA was isolated from cultured rat astrocytes using the RNAeasy mini kit (Qiagen, Hilden, Germany) according to the manufacturers´ instructions. RNA was quantified using the NanoDrop1000 System (Thermo Scientific, Wilmington, USA) and first strand cDNA was synthesized by using the QuantiTect Reverse Transcription Kit (Qiagen, Hilden, Germany) according to the manufacturers´ instructions. Gene expression levels were quantified by measuring Bryt^®^ Green (Promega, Mannheim, Germany) fluorescence intensities using the 7500 real-time PCR system (Applied Biosystems®, Life Technologies, Darmstadt, Germany). PCR-primer sequences used for quantitative realtime-PCR are summarized in Table 1. Data for each gene and sample were produced in duplicate and mean values of cycle numbers for the target amplification were subtracted from the mean of cycle numbers of the house-keeping gene (Hprt1) of the respective sample and taken to the power of 2. This value represents mRNA expression levels of the respective target gene in relation to Hprt1 expression. Relative mRNA expression changes are expressed as log_2_-ratios.

### Quantitative measurement of astrocyte proliferation

Astrocyte proliferation was measured as described earlier^11^. In brief, approximately 12000 cells were seeded in each cavity of a 24 well and treated as indicated or were left untreated. For experiments using Dharmacon® miRNA inhibitors, cells were seeded on 24 well plates 6 h after transfection. At the end of the experimental treatment cells were fixed using paraformaldehyde (4%, 5 min, RT) and DNA was stained using Hoechst34580 (1:5000, 15 min, RT). Cells were washed thrice with PBS and Hoechst34580 fluorescence was detected by a fluorimeter (Fluoroscan Ascent FL, Thermo Scientific, Darmstadt, Germany) using excitation/emission wavelengths of 380 and 460 nm, respectively. For each experimental condition per astrocyte seeding measurements were performed on 10–12 independent wells of the 24 well plate. Mean fluorescence intensities were calculated and corrected for background fluorescence of cells not stained with Hoechst34580. Proliferation in experimentally-treated cells was expressed as fold of the respective control condition as indicated.

### RNA isolation and cDNA synthesis for miRNA analysis by using miQPCR

To isolate miRNA-containing RNAs, cells were lysed and homogenized with Acid Phenol (Ambion®, Life Technologies, Darmstadt, Germany) and RNA was isolated using the miRVana RNA isolation kit (Ambion®, Life Technologies, Darmstadt, Germany) according to the instructions provided by the manufacturer. RNA concentration was measured using the Nanodrop 1000 (Thermo Scientific, Waltham, USA) and RNA integrity was assessed using the Bioanalyzer (Agilent, Santa Clara, USA).

cDNA synthesis and primer design (Tab. 2) for the analysis of miRNA expression, profiling by miQPCR was carried out as previously described^31^. In brief, 10 ng of total RNA was used for cDNA synthesis and a fraction of the synthesized cDNA (equivalent to 1% of final cDNA volume) was used for individual qPCR assays. Primer design was performed as described in^31^, miRNA specific primers used in this study are listed in Table 2. qPCR assays were performed in a 96 well format and run on a ViiA7 thermocycler (Applied Biosystems®, Life Technologies, Darmstadt, Germany) using Bryt^®^ Green (Promega, Mannheim, Germany) miRNA amplification parameters were as follows: 95 °C for 10 minutes (1 cycle), 95 °C for 15 seconds and 60 °C for 35 seconds (50 cycles), followed by melting curve analysis. qPCR data were analyzed by qBase software v.1.3.5^46^.

### Affymetrix rat gene microarray and Agilent rat miRNA microarray analysis

3 biological replicas of untreated (48 h) and NH_4_Cl (5 mmol/l, 48 h)-exposed cultured rat astrocytes were hybridized to rat Affymetrix GeneChip 1.0 ST arrays. cDNA synthesis and labeling were performed according to the Affymetrix protocol. Hybridization and washing were carried out in a SciGene 777 Microarray Oven (50 rpm, 45 °C for 16 h) and in an Affymetrix GeneChip 450 Washer respectively. Microarrays scanning and acquisition of signal intensities were performed on the Affymetrix GeneChip Scanner GCS3000 System. Microarray data analysis of the Affymetrix arrays was performed by using the AltAnalyze software^47^ by running the default settings. Specifically microarrays were normalized by using RMA (Robust Microarray Analysis) with a 2-fold cut-off and a P-value <0.05. Hierarchical clustering of the biological replicas was performed by using the AltAnalyze software v. 2.0.8.1.

Quality control, hybridization and analysis of RNA to Agilent rat array was carried out as follows. Total RNA was quality controlled on Agilent Bioanalyzer, quantification was carried out using Invitrogen Qubit RNA (Invitrogen™, Life Technologies, Darmstadt, Germany) and the RNA concentration was normalized to 200 ng input.

The one-color (Cy3) labeling reaction was carried using Agilent miRNA labeling kit as per protocol of the manufacturer, except that DMSO was not purified on column and samples were speed-vac concentrated after labeling. Hybridization was performed using Agilent miRNA Hyb kit and samples were hybridized on Agilent miRNA v19 8 × 60k microarrays for 20 hours then scanned using Agilent DNA High-Resolution Scanner and data extracted using Feature Extraction 10.7.3.1. Microarray data generated by the extraction features are in a median normalized format. Initial clustering of the unpaired samples was performed with MultiExperiment View (MeV)^48^. miRNA expression changes between untreated and NH_4_Cl (5 mmol/l, 48 h)-exposed astrocytes of the individual astrocyte preparation were larger than the difference arising from the respective biological replicas. In order to reduce noise and to identify miRNAs species modulated by ammonia with higher probability Log_2_ ratios of the paired samples were calculated.

### Transfection of rat primary astrocytes with miRNA inhibitors

Inhibition of rno-miR-31a-5p, -221-3p, -221-5p, -222-3p, -326-3p and -365-3p in cultured astrocytes was performed using miRIDIAN® miRNA hairpin inhibitors (Dharmacon, GE Healthcare Life Sciences, Munich, Germany). Inhibitors were diluted at a final concentration of 40 nmol/l and transfected by using Lipofectamine 2000 reagent (Invitrogen^TM^, Life Technologies, Darmstadt, Germany) following the protocol of the manufacturer. 6 h after transfection, cell culture media was removed and cells were washed using DMEM (1000 mg/l D-Glucose, GlutaMAX^TM^) containing 10% FCS following cultivation for another 48 h.

### Analysis of cell viability

Cell viability was analyzed by measuring lactate-dehydrogenase (LDH)-activity in the cell culture supernatant as described recently^3^. Dead and damaged cells were identified by propidium iodide (PI) staining (25 μmol/l, 20 min). For a positive control, cells were treated with digitonin (160 nmol/l, 20 min) to permeabilize the cell membrane. Apoptotic cells were identified by terminal deoxynucleotidyl transferase dUTP nick end labeling according to the instructions of the manufacturer (TUNEL, Roche, Mannheim, Germany).

### Online databases

miRNA sequences were acquired from miRBase database (http://www.mirbase.org; v21)^49^. Melting temperatures for miRNA primers were calculated with the online tool Tm calculator (Applied Biosystems, http://www6.appliedbiosystems.com/support/techtools/ calc/index.cfm). Primers for mRNA amplification were assessed by Oligo Analyzer (http://eu.idtdna. com/calc/analyzer) with respect to their secondary structure, possible self-dimers and primer duplexes. miRNA target prediction was performed by either querying miRWALK (http://www.umm.uni-heidelberg.de/apps/zmf/mirwalk/) or RNA22 (https://cm.jefferson.edu/data-tools-downloads/). Venn diagrams and intersection between gene lists were calculated with the on-line tool Venny (http://www.bioinfogp.cnb.csic.es/tools/venny).

### Statistical analysis of results

Experiments on cultured astrocytes were carried out with at least 3 independent astrocyte preparations. Results are presented as mean values ± SEM. Statistical analysis was performed using two-sided Student´s *t*-test or one way analysis of variance (ANOVA) followed by Tukey´s or Dunnet´s multiple comparison *post hoc* tests where appropriate (GraphPad Prism; GraphPad, La Jolla, USA; Excel for Windows; Microsoft, Redmond, USA). A P-value ≤ 0.05 was considered statistically significant.

## Additional Information

**How to cite this article**: Oenarto, J. *et al*. Ammonia-induced miRNA expression changes in cultured rat astrocytes. *Sci. Rep*. **6**, 18493; doi: 10.1038/srep18493 (2016).

## Supplementary Material

Supplementary Information

## Figures and Tables

**Table 1:** Primer sequences used for realtime-PCR analysis.

Name	Description	Forward Primer	Reverse Primer
Abca5	ATP-binding cassette sub-family A, member 5	TGTCCAGTCTCTCTCACAGTCC	ATTCTTCAATGGCAAAAGTGTG
Adm2	adrenomedullin 2	GTCTGGCAGCCTGGGTAAG	CGCTGGAAGGAATCTTGG
Artn	artemin	AGAAGAGGGTGGGGAAACAG	CAGTGGGACAATGCAGTAGG
Creb3I1	cAMP responsive element binding protein 3-like 1	AGTGTCTACGCAGCCAGTCA	GCCTGCCCCATCATCATA
Cth	cystathionine g-lyase	CGAAGACCTGGGTCAAGC	AGAAGGTCTGGCCCCTTG
Gadd45α	growth arrest and DNA-damage-inducible 45a	TCTGTTGCGAGAACGACATC	TGTGATGAATGTGGGTTCGT
Galnt3	polypeptide N-acetylgalactosaminyltransferase 3	GGATGGACGAGTACAAGGAAA	TCTTTTTGAAAGATCACCAAATGAT
Gmds	GDP-mannose 4,6-dehydratase	GAAATACTACCGACCGACTGAAG	GTTTCCAGTTCAGTTTCTGCTG
Herpud1	homocysteine-inducible, endoplasmic reticulum stress-inducible, ubiquitin-like domain member 1	GAACCTTCCTCCCTCTGGAT	CCTTGGAAAGTCTGCTGGAC
HO-1	heme oxygenase 1	CGGCCCTGGAAGAGGAGATAG	CGATGCTCGGGAAGGTGAAAA
Hprt1	hypoxanthine ribosyl transferase 1	TGCTCGAGATGTCATGAAGGA	CAGAGGGCCACAATGTGATG
Ifit2	interferon-induced protein with tetratricopeptide repeats 2	CACCGACACCACAAACAGTC	GTGGCATTTGAGCTGCTGTA
Il6	interleukin 6	GCCAGAGTCATTCAGAGCAA	CATTGGAAGTTGGGGTAGGA
Myc	v-myc avian myelocytomatosis viral oncogene homolog	GCTCCTCGCGTTATTTGAAG	GCATCGTCGTGACTGTCG
Nr4a1	nuclear receptor subfamily 4, group A, member 1	CATCTTCTTCCTCGTCCTCG	AACTGCTCAGTCCATACCCG
Serpine1	serpin peptidase inhibitor, clade E, member 1	TCTGGGAAAGGGTTCACTTTACC	GACACGCCATAGGGAGAGAAG
Shmt2	serine hydroxymethyltransferase 2	GGTGAAGCGCAAGACTGC	GCCAAACGCTGACTTGTTTC
Slc1a4	solute carrier family 1, member 4	ACGCGGGACAGATTTTCAC	ACACCCGCTGCTCCAAC
Slc1a5	solute carrier family 1, member 5	TCCTCTTTACCCGCAAAAAC	CCACACCATTCTTCTCCTCTAC
Slc6a9	solute carrier family 6, member 9	CTCTCACACTGCTTCCCATC	TCATTCCCCACCTCATCCAC
Tlcd1	TLC domain containing 1	ATCTGGGATCTGGGCTCTG	GCAAATAGCCACAAACTGACC
Wars	tryptophanyl-tRNA synthetase	GCAAAATTGACAAGGAGCTGA	TTCATAGGCATCCAGAATTTGA
Yars	tyrosyl-tRNA synthetase	CCCAGTTTGGCGGTATTG	GCCAAGTGTGGGGAGATACT

List of qPCR primers for the amplification of mRNA target genes used in this study. Primer sequences are given in 5′ to 3′ direction.

**Table 2:** Primer sequences used for miQPCR analysis.

Name	Primer sequence	Tm (°C)
rno-miR-221-5P	GGCATACAATGTAGATTTCG	51.80
rno-miR-222-3P	CTACATCTGGCTACTGGGTG	54.24
rno-miR-135a-5p	TGGCTTTTTATTCCTATGTGAG	54.81
rno-miR-26a-5p	AAGTAATCCAGGATAGGCTG	52.11
rno-miR-425-5p	CGATCACTCCCGTTGAG	54.12
rno-miR-30e-5p	AACATCCTTGACTGGAAGG	53.40
rno-miR-652-3p	CGCCACTAGGGTTGTGG	56.90
rno-miR-135b-5p	GGCTTTTCATTCCTATGTGAG	55.00
rno-miR-31a-5p	GATGCTGGCATAGCTGG	54.47
rno-miR-125a-5p	TGAGACCCTTTAACCTGTGAG	55.41
rno-miR-193-3p	GCCTACAAAGTCCCAGTG	52.31
rno-miR-221-3p	TTGTCTGCTGGGTTTCG	54.91
rno-miR-30b-5p	TAAACATCCTACACTCAGCTG	51.63
rno-miR-326-3P	GGCCCTTCCTCCAGTG	55.70
rno-miR-145-5P	TTCCCAGGAATCCCTG	53.19
rno-miR-365-3p	GCCCCTAAAAATCCTTATG	52.52
Upm2A	CCCAGTTATGGCCGTTTA	55.50

List of miRNA specific qPCR primers and of the Universal reverse primer (Upm2A) used in this study. Primer sequences are given in 5′ to 3′ direction.
