# Supplementary material for: Ammonia-induced miRNA expression changes in cultured rat astrocytes
Source: Sci Rep. 2016 Jan 12;6:18493. doi: 10.1038/srep18493 (PMC4709596; doi:10.1038/srep18493)
Supplement: Supplementary Information [file srep18493-s1.pdf]

Ammonia-induced miRNA expression changes in cultured rat astrocytes

Jessica Oenarto, Ayse Karababa, Mirco Castoldi, Hans J. Bidmon, Boris Görg and Dieter Häussinger

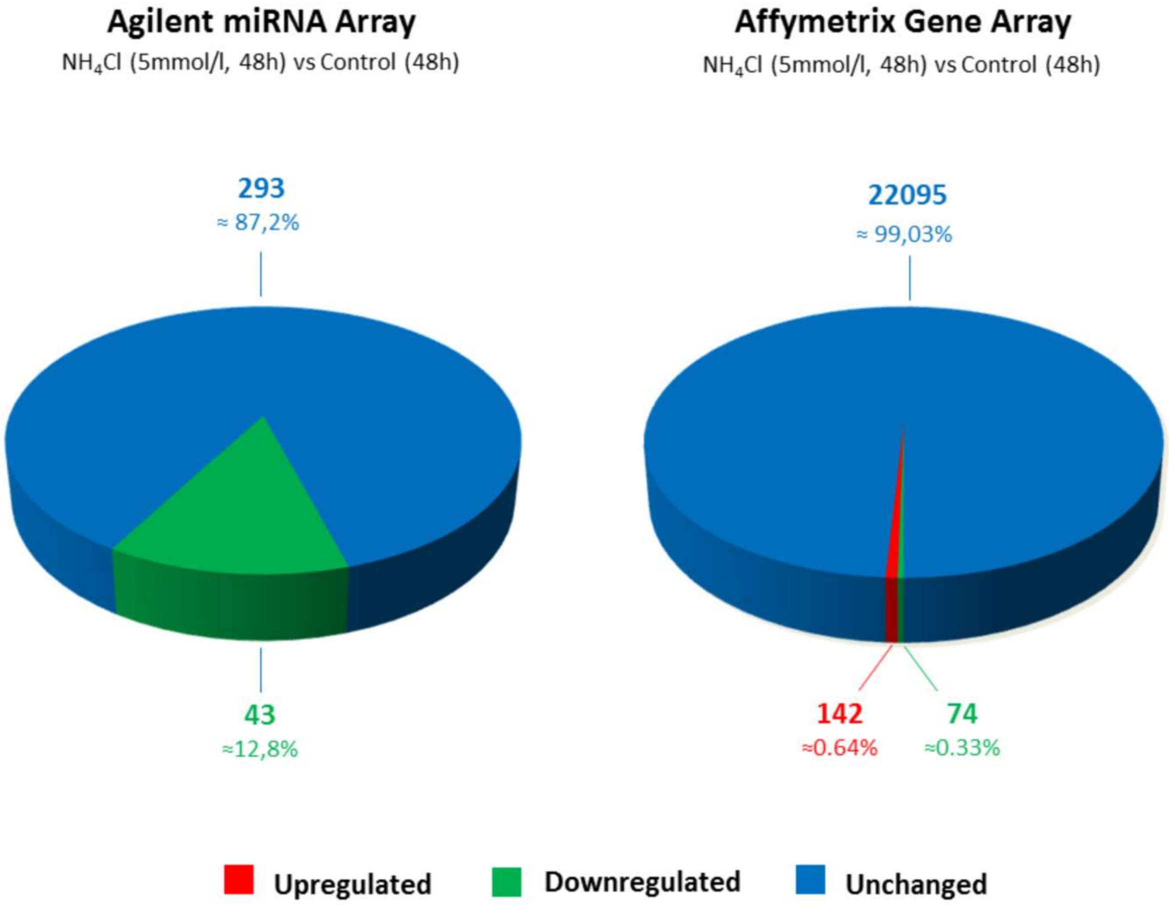

Supplementary Figure 1

Ammonia-induced miRNA expression changes in cultured rat astrocytes

Jessica Oenarto, Ayse Karababa, Mirco Castoldi, Hans J. Bidmon, Boris Görg and Dieter Häussinger

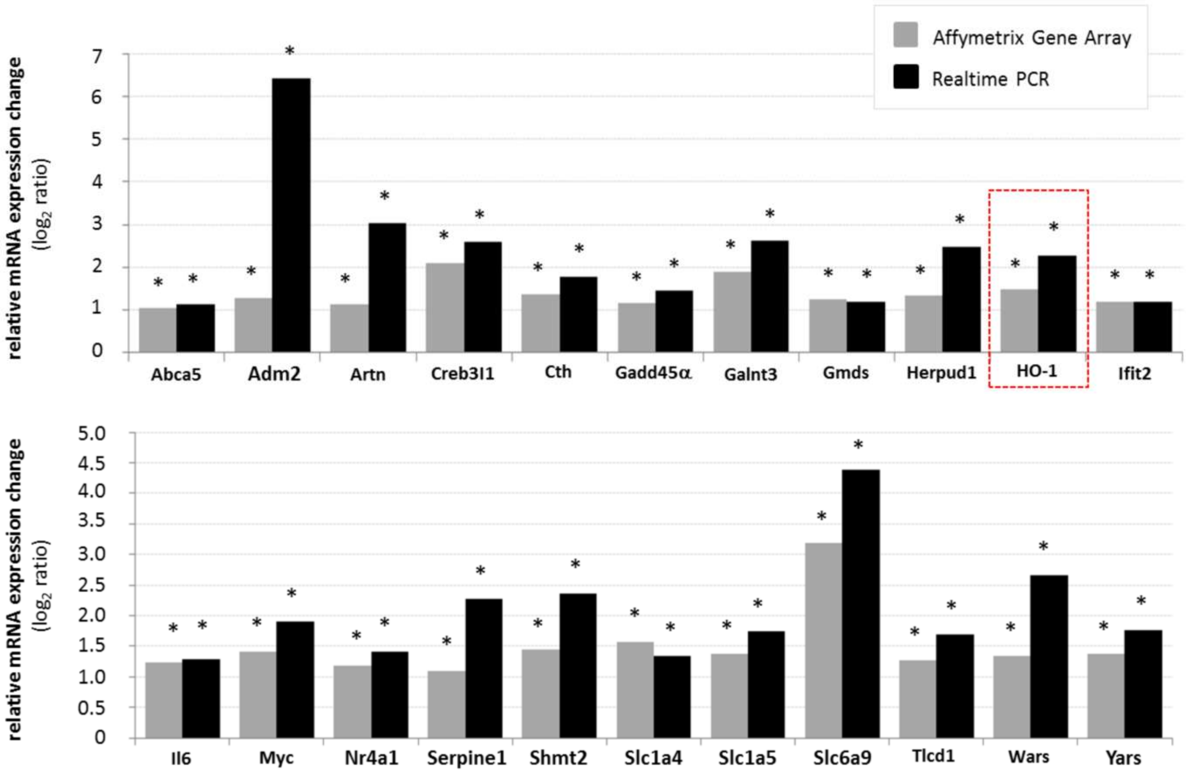

Supplementary Figure 2

Ammonia-induced miRNA expression changes in cultured rat astrocytes

Jessica Oenarto, Ayse Karababa, Mirco Castoldi, Hans J. Bidmon, Boris Görg and Dieter Häussinger

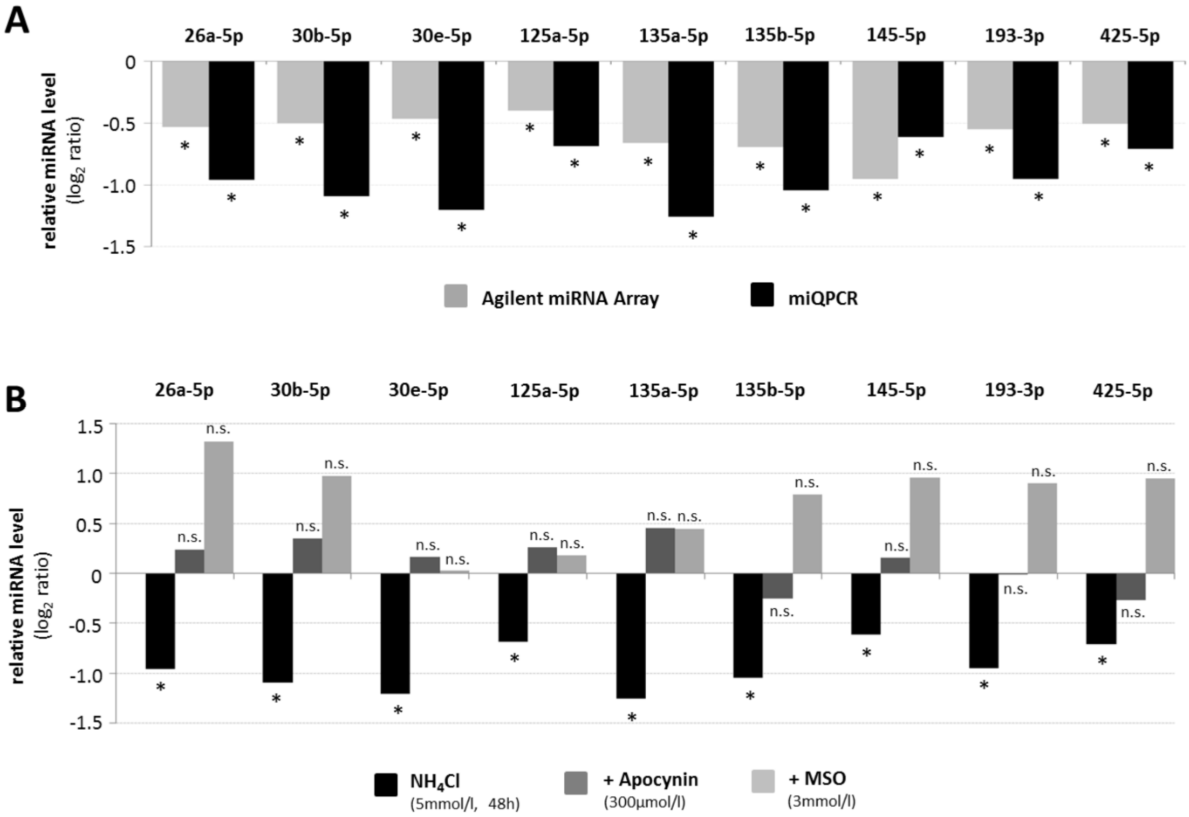

Supplementary Figure 3

Ammonia-induced miRNA expression changes in cultured rat astrocytes

Jessica Oenarto, Ayse Karababa, Mirco Castoldi, Hans J. Bidmon, Boris Görg and Dieter Häussinger

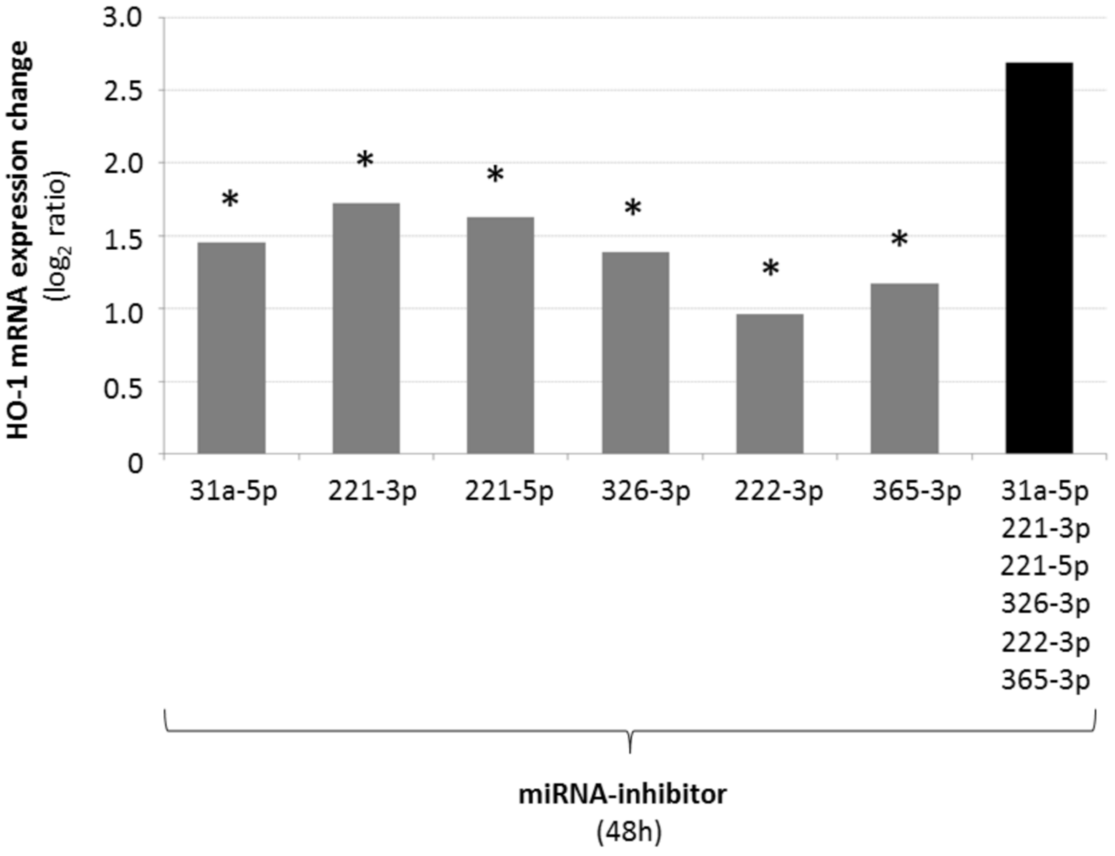

Supplementary Figure 4

Ammonia-induced miRNA expression changes in cultured rat astrocytes

Jessica Oenarto, Ayse Karababa, Mirco Castoldi, Hans J. Bidmon, Boris Görg and Dieter Häussinger

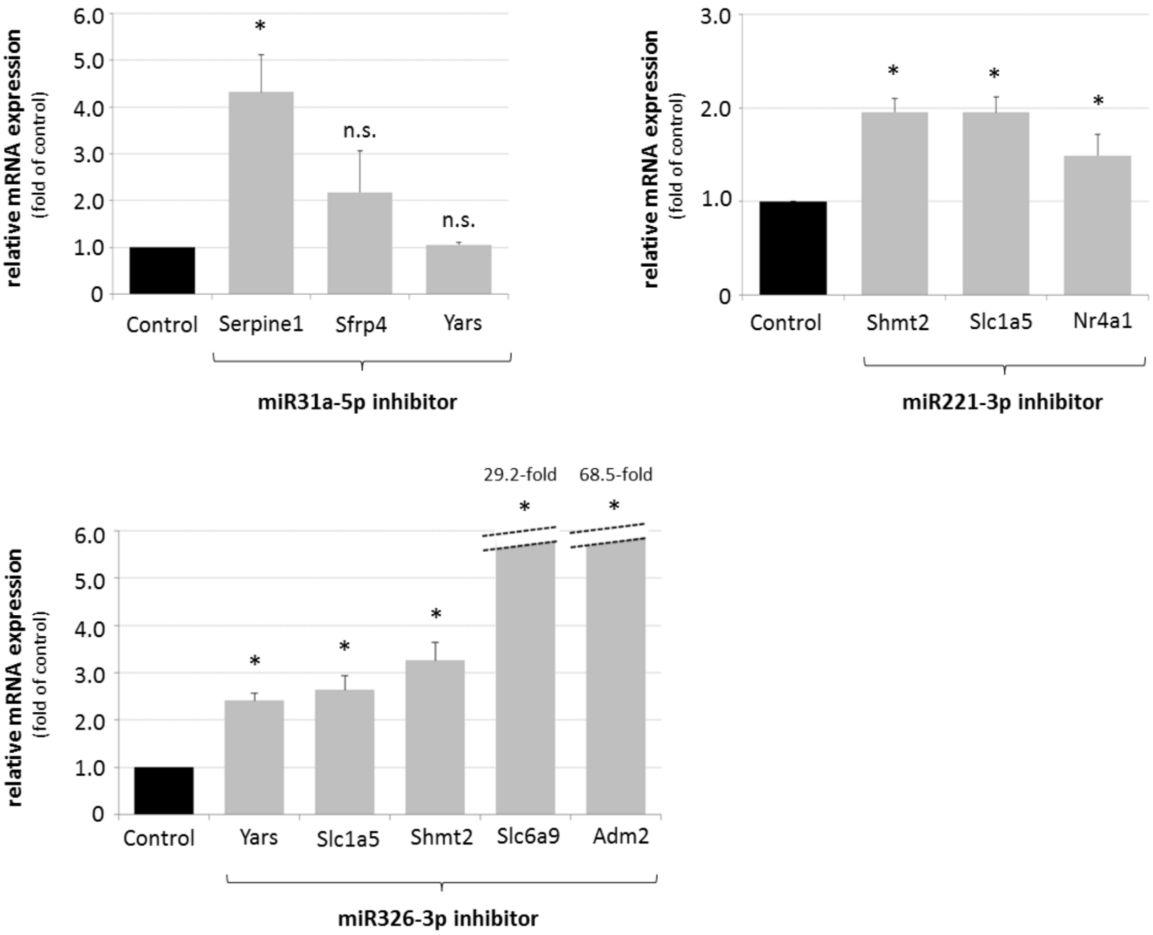

Supplementary Figure 5

Ammonia-induced miRNA expression changes in cultured rat astrocytes

Jessica Oenarto, Ayse Karababa, Mirco Castoldi, Hans J. Bidmon, Boris Görg and Dieter Häussinger

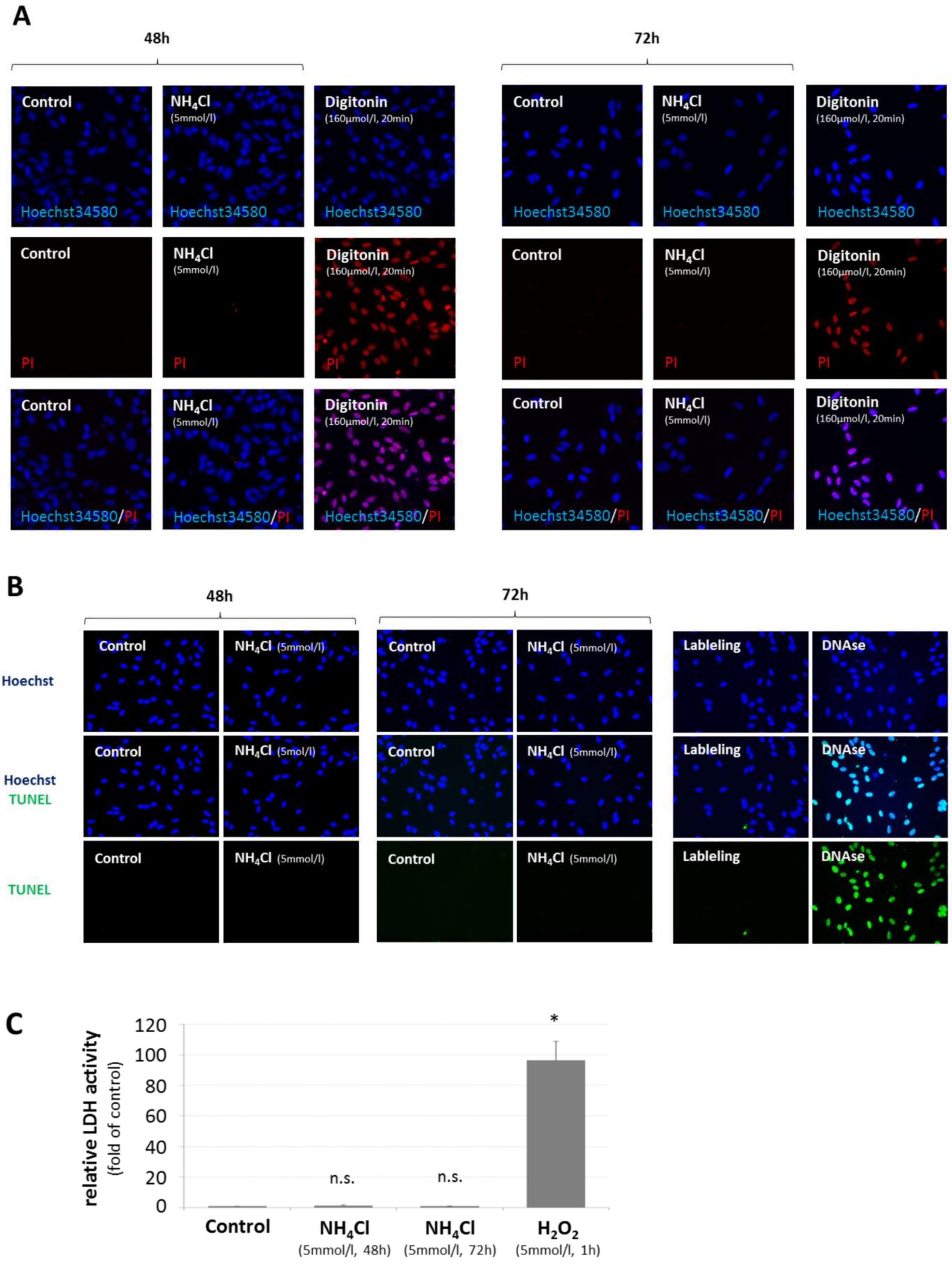

Supplementary Figure 6

Ammonia-induced miRNA expression changes in cultured rat astrocytes

Jessica Oenarto, Ayse Karababa, Mirco Castoldi, Hans J. Bidmon, Boris Görg and Dieter Häussinger

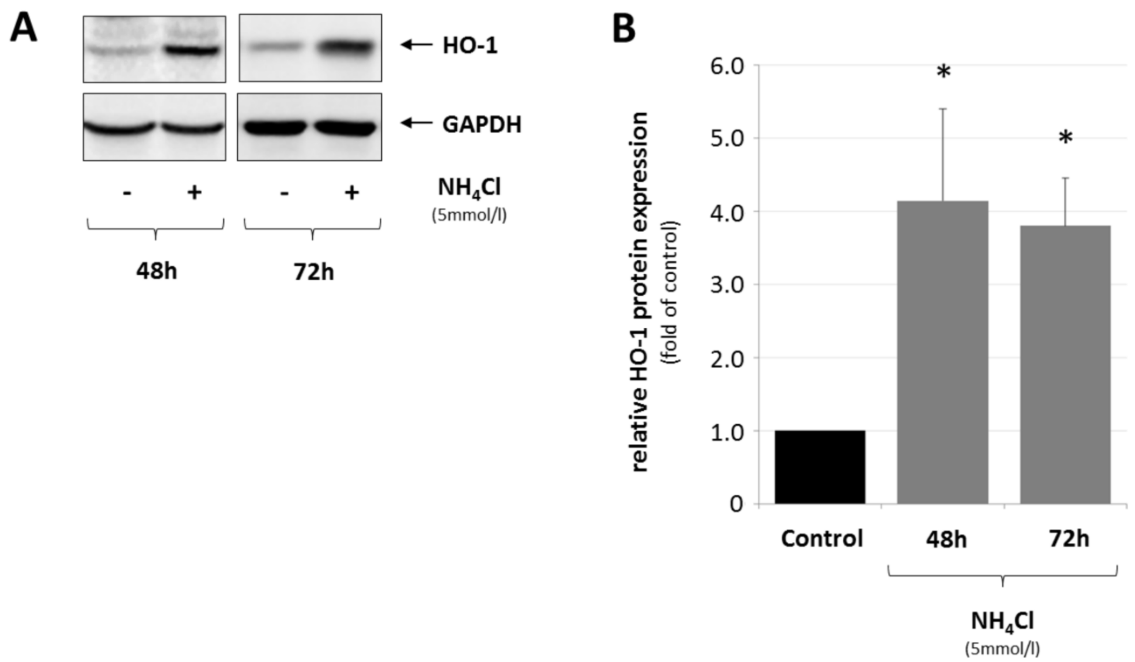

Supplementary Figure 7

Ammonia-induced miRNA expression changes in cultured rat astrocytes

Jessica Oenarto, Ayse Karababa, Mirco Castoldi, Hans J. Bidmon, Boris Görg and Dieter Häussinger

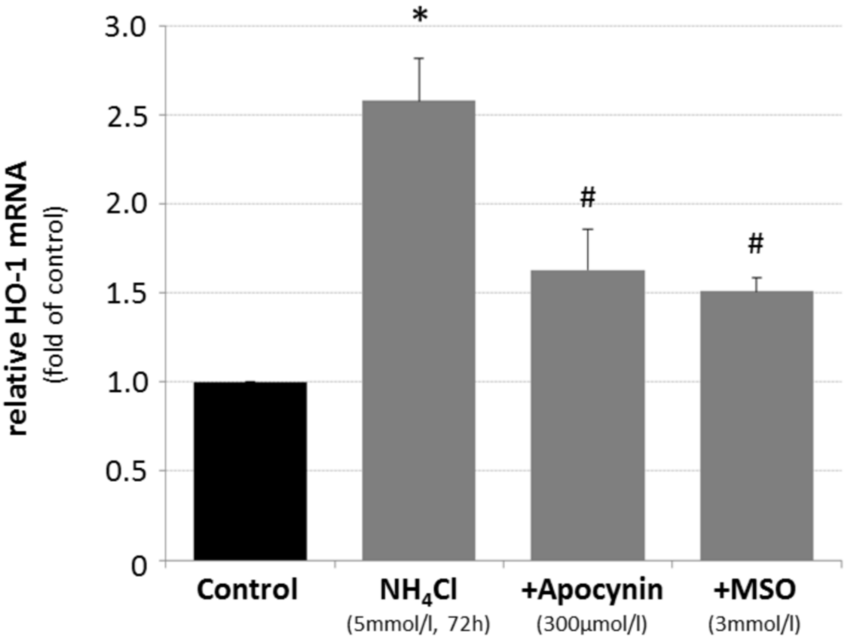

Supplementary Figure 8

Ammonia-induced miRNA expression changes in cultured rat astrocytes

Jessica Oenarto, Ayse Karababa, Mirco Castoldi, Hans J. Bidmon, Boris Görg and Dieter Häussinger

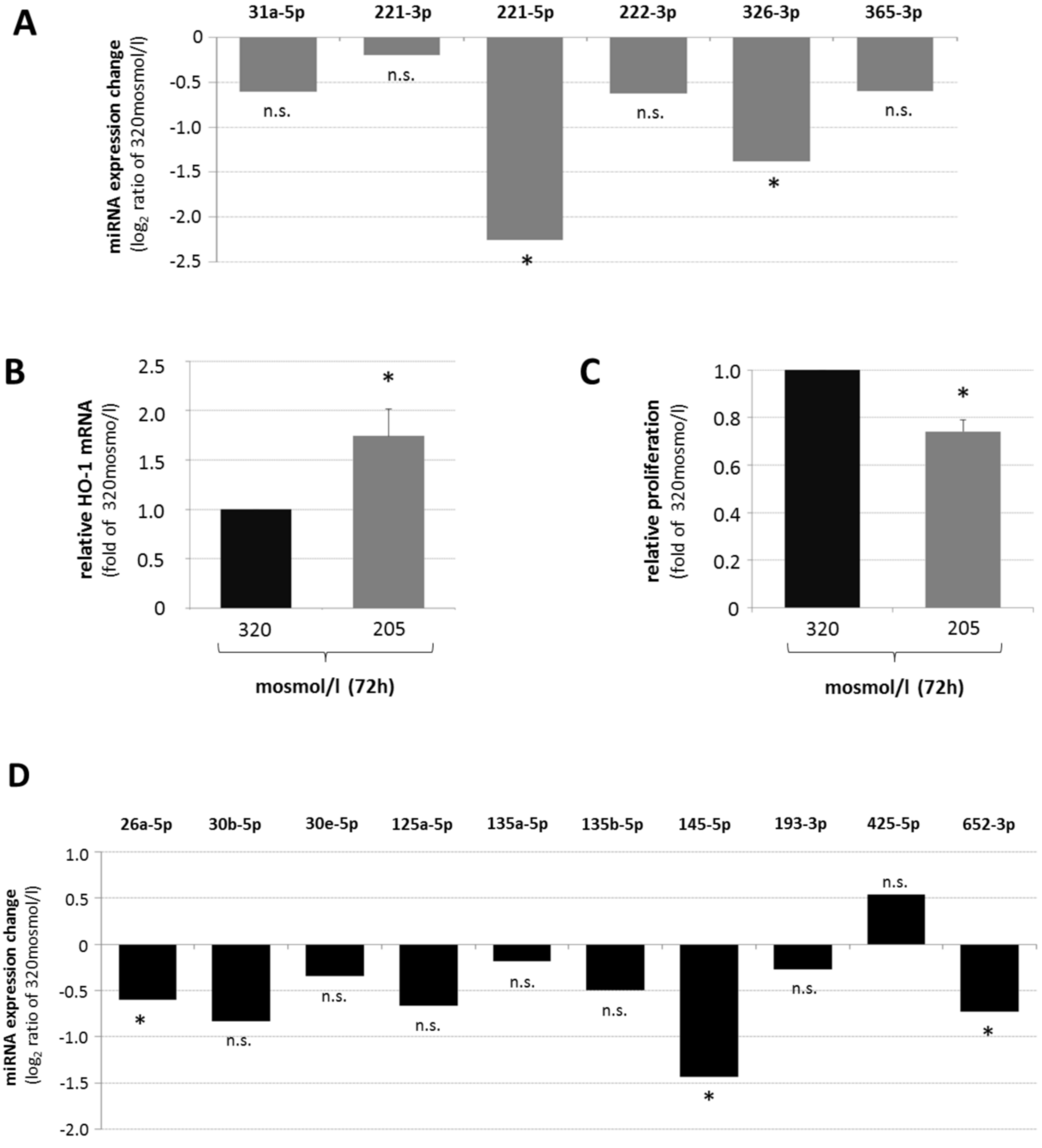

Supplementary Figure 9

Ammonia-induced miRNA expression changes in cultured rat astrocytes

Jessica Oenarto, Ayse Karababa, Mirco Castoldi, Hans J. Bidmon, Boris Görg and Dieter Häussinger

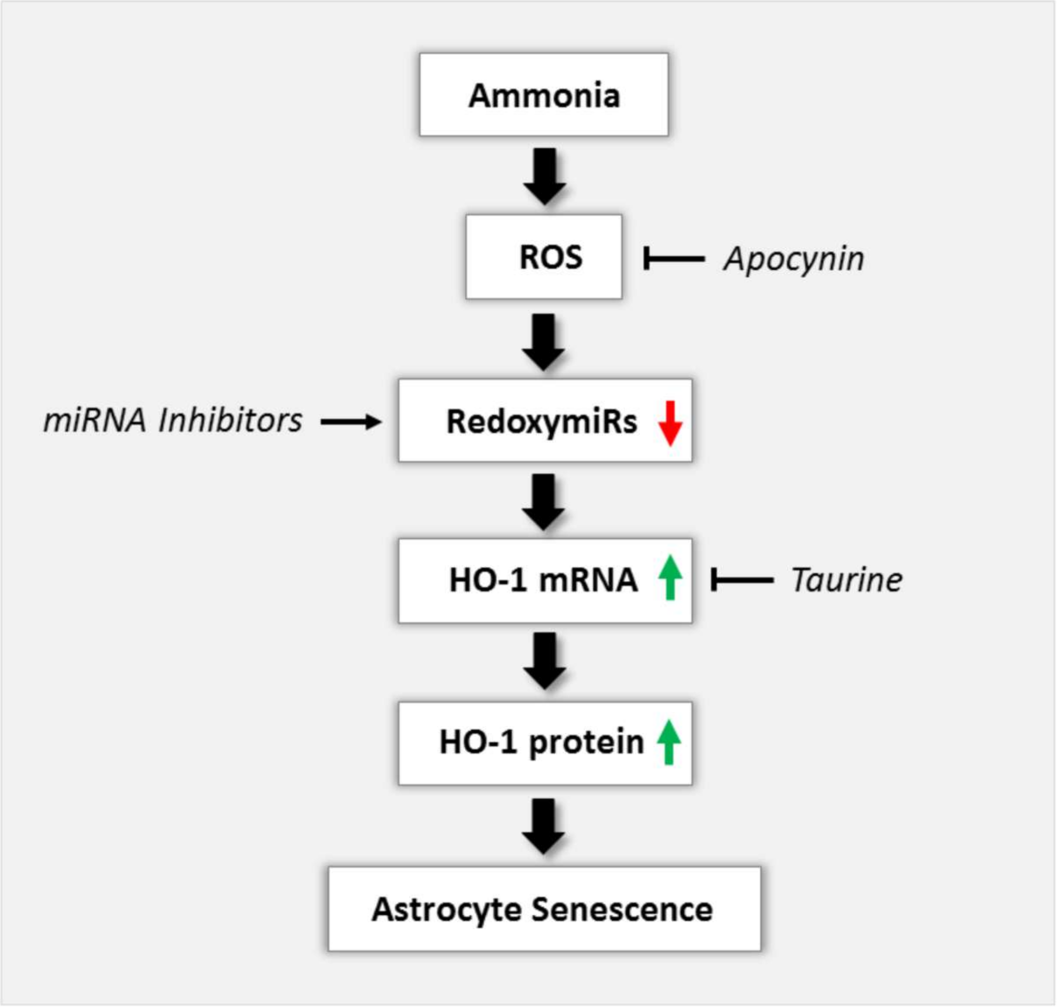

Supplementary Figure 10

Ammonia-induced miRNA expression changes in cultured rat astrocytes

Jessica Oenarto, Ayse Karababa, Mirco Castoldi, Hans J. Bidmon, Boris Görg and Dieter Häussinger

Supplementary Tables

Supplementary Table 1

miRNA name (rno-miR)	miRNA sequence	miRNA seed	HO-1 region	Seed length	Seed start	Seed end
326-3p	CCTCTGGGCCCTTCCTCCAGT	CCTCTGGG	3´UTR	8 nts	1001	1008
221-3p	AGCTACATTGTCTGCTGGGTTTC	GCTACAT	3´UTR	7 nts	1025	1031
222-3p	AGCTACATCTGGCTACTGGGT	GCTACAT	3´UTR	7 nts	1025	1031
31a-5p	AGGCAAGATGCTGGCATAGCTG	AGGCAAGAT	3´UTR	9 nts	1051	1059
365-5p	TAATGCCCCTAAAAATCCTTAT	TAATGCC	3´UTR	7 nts	1533	1539

Supplementary Table 2

miRNA-221-5p sequence	miRNA/HO-1 3´UTR alignment	HO-1 region	Binding start
ACCTGGCATACAATGTAGATTTC	CAGCTCGACAGCATGTCCCAGGA || ||| |:| ||||| CTTTAGATGTAACATACGGTCCA	CDS	129
ACCTGGCATACAATGTAGATTTC	TGACCTCTCAGGG--GGTCAGGT ||	|:||||| CTTTAGATGTAACATACGGTCCA	CDS	533
ACCTGGCATACAATGTAGATTTC	TTTGTCT-CTCTGGAATGGAAGGA<br>:|||	|| ||| ||| CTTTAGATGTAAC-ATACGGTCCA	3´UTR	1124
ACCTGGCATACAATGTAGATTTC	GCTGTCT--TTTGAGGGGTGGGT<br>:||| |||	::||| CTTTAGATGTAACATACGGTCCA	3´UTR	1460

Ammonia-induced miRNA expression changes in cultured rat astrocytes

Jessica Oenarto, Ayse Karababa, Mirco Castoldi, Hans J. Bidmon, Boris Görg and Dieter Häussinger

Supplementary Figure Legends

Supplementary Figure 1: Overview on miRNA and gene expression changes in ammonia-exposed cultured rat astrocytes. Measurement of miRNA and gene expression levels in cultured rat astrocytes by Agilent miRNA array or Affymetrix rat gene array. miRNA and mRNA expression level changes are illustrated in a pie chart comparing controls with NH_4Cl (5mmol/l, 48h)-exposed cultured rat astrocytes. Data are from 3 independent experiments.

Supplementary Figure 2: Validation of ammonia-induced gene expression changes in cultured rat astrocytes. Cultured rat astrocytes where exposed to NH_4Cl (5mmol/l) or were left untreated (control) for 48h before RNA was isolated and analysed by Affymetrix Gene Array. Relative gene expression changes of at least two-fold and a p-value ≤ 0.05 were considered statistically significant. 22 out of 142 significantly upregulated genes in NH_4Cl-treated astrocytes were selected for validation by realtime-PCR. * statistically significantly different compared to untreated astrocytes. n.s. not significantly different compared to untreated astrocytes. Data are represented as log_2 ratios from 4-12 independent experiments.

Supplementary Figure 3: Validation of miRNA expression changes and analysis of their dependence on glutamine synthesis and NADPH-oxidase activation in ammonia-exposed astrocytes. (A/B) Cultured rat astrocytes where exposed to NH_4Cl (5mmol/l) or were left untreated (control) for 48h before RNA was isolated and analysed for miRNA expression changes by Agilent miRNA Array **(A)** or miQPCR **(A/B)** as described in materials and methods. **(A)** Validation of miRNA expression changes by miQPCR. **(B)** Glutamine synthetase- and NADPH-oxidase dependence of miRNA expression changes in NH_4Cl (5mmol/l, 48h)-exposed astrocytes. Where indicated astrocytes where treated with MSO (3mmol/l) or apocynin (300µmol/l). * statistically significantly different to the respective controls (untreated or inhibitor-treated). n.s.: not significantly different to the respective control (untreated or inhibitor-treated). Data are represented as log_2 ratios from 3-5 independent experiments.

Ammonia-induced miRNA expression changes in cultured rat astrocytes

Jessica Oenarto, Ayse Karababa, Mirco Castoldi, Hans J. Bidmon, Boris Görg and Dieter Häussinger

Supplementary Figure 4: Combined inhibition of miRNAs predicted to target HO-1 and being downregulated in ammonia-exposed astrocytes elevate HO-1 mRNA levels in a synergistic manner. Cultured astrocytes were transfected either with individual miRNA-inhibitors targeting rno-miR-31a-5p, 221-3p, 221-5p, 326-3p, 222-3p or 365-3p or with a mixture of all (each 40nmol/l, 48h). Total RNA was isolated and HO-1 mRNA levels were analysed by realtime-PCR. * statistically significantly different compared to astrocytes simultaneously treated with all of the indicated miRNA-inhibitors. Data are represented as log_2 ratios from 5-9 independent experiments.

Supplementary Figure 5: Effect of inhibition of miRNAs rno-miR-31a-5p, -221-3p, -221-5p or -326-3p on expression levels of predicted mRNA targets. (A-C) Cultured astrocytes were transfected either with inhibitors specifically targeting rno-miR-31a-5p, -221-3p, -221-5p or -326-3p or without inhibitor (control) before RNA was isolated and mRNA levels of predicted target mRNAs were analysed by realtime-PCR. * statistically significantly different compared to control-transfected astrocytes. n.s.: not significantly different to control-transfected astrocytes. Data are from 3-9 independent experiments.

Supplementary Figure 6: Effect of NH_4Cl on astrocyte viability. Astrocytes were either treated with 5mmol/l NH_4Cl for 48 or 72h, H_2O_2 (5mmol/l, 1h) or were left untreated for 1, 48 or 72h (control) , respectively. (A) Detection of dead and damaged cells by propidium-iodide (PI) staining. For positive control, cells were treated with digitonin (160µmol/l, 20min) to permeabilize the cell membrane. Nuklei were counterstained using Hoechst34580. (B) Analysis of apoptosis in NH_4Cl (5mmol/l, 48 or 72h)-treated cultured astrocytes by terminal deoxynucleotidyl transferase X-dUTP nick-end labeling *(TUNEL)* assay. For negative control (“Labeling”), TUNEL reaction was carried out in the absence of deoxynucleotidyl transferase. For positive control („DNAse-treated“), DNA strand breaks were induced by DNAse treatment (1.500U/ml DNAse I, 1h) according to the manufacturers´ instructions. Nuclei were counterstained using Hoechst34580. (C) Lactate dehydrogenase (LDH) activity in the supernatant of cultured astrocytes. LDH activity in NH_4Cl- or H_2O_2-treated is given relative to the respective untreated control. *: statistically significantly different compared to untreated controls. n.s.: not statistically significantly different compared to untreated controls. Data are from 3-5 independent experiments.

Ammonia-induced miRNA expression changes in cultured rat astrocytes

Jessica Oenarto, Ayse Karababa, Mirco Castoldi, Hans J. Bidmon, Boris Görg and Dieter Häussinger

Supplementary Figure 7: Effect of NH_4Cl on HO-1 protein expression. Astrocytes were either treated with 5mmol/l NH_4Cl or were left untreated (control) for 48 or 72h, respectively. Protein was isolated and HO-1 was detected by Western-blot analysis (A). (B) Densitometric analysis of HO-1 protein expression. HO-1 protein expression in NH_4Cl-treated astrocytes is given relative to the respective control.

Supplementary Figure 8: Effects of apocynin and MSO on HO-1 mRNA expression in cultured astrocytes. Cultured astrocytes were exposed to NH_4Cl (5mmol/l) or were left untreated (control) for 72h before RNA was isolated and analysed for HO-1 mRNA expression by realtime-PCR. Where indicated astrocytes were treated with MSO (3mmol/l) or apocynin (300µmol/l). HO-1 mRNA expression in NH_4Cl (5mmol/l)-exposed astrocytes is given relative to the respective control (untreated or inhibitor treated). * statistically significantly different to control. # statistically significantly different to NH_4Cl-treated astrocytes. Data are from 3-14 independent experiments.

Supplementary Figure 9: Effect of hypoosmotic astrocyte swelling on miRNA and HO-1 mRNA expression and astrocyte proliferation. Cultured astrocytes where exposed to normoosmotic (320mosmol/l) or hypoosmotic (205mosmol/l) cell culture media for 72h. (A) Quantification of HO-1 targeting miRNAs (A) and further miRNA species not predicted to target HO-1 (D) by miQPCR. Data are are represented as log_2 ratios. (B) Quantification of HO-1 mRNA expression levels by realtime-PCR. miRNA/mRNA expression levels found in astrocytes treated with hypoosmotic cell culture media (205mosmol/l) are given relative to normoosmotic controls (320mosmol/l). (C) Measurement of astrocyte proliferation. DNA content was quantified by fluorimetric detection of Hoechst34580 fluorescence as described in materials and methods and fluorescence intensities found in astrocytes treated with hypoosmotic cell culture media (205mosmol/l) is given relative to normoosmotic controls (320mosmol/l). * statistically significantly different to normoosmotic controls. n.s. : not statistically signficantly different to normoosmotic controls. Data are from 3-6 independent experiments.

Supplementary Figure 10: Scheme on the proposed signaling pathways underlying ammonia-induced astrocyte senescence. Ammonia increases the formation of reactive oxygen species (ROS) through activation of NADPH-oxidase. ROS upregulates heme oxygenase 1 (HO-1) mRNA through downregulation of HO-1 targeting redoximiRs. Upregulation of HO-1 protein induces astrocyte senescence by a hitherto unknown mechanism.

Ammonia-induced miRNA expression changes in cultured rat astrocytes

Jessica Oenarto, Ayse Karababa, Mirco Castoldi, Hans J. Bidmon, Boris Görg and Dieter Häussinger

Supplementary Table Legends

Supplementary Table 1: Putative miRNA binding site within HO-1 3´UTR as predicted by miRWALK. 3´UTR: 3´UTR untranslated region.

Supplementary Table 2: Putative miR-221-5p binding site within HO-1 mRNA as predicted by RNA22. CDS: coding sequence, 3´UTR: 3´UTR untranslated region.
